# Infertility in Men with Spinal Cord Injury: Research and Treatment

**DOI:** 10.6064/2012/578257

**Published:** 2012-11-25

**Authors:** Nancy L. Brackett

**Affiliations:** Lois Pope Life Center, The Miami Project to Cure Paralysis, University of Miami Miller School of Medicine, Room 2-17, 1095 NW 14th Terrace, Miami, FL 33136, USA

## Abstract

Spinal cord injury (SCI) occurs most often to young men. Following SCI, most men are infertile due to a combination of erectile dysfunction, ejaculatory dysfunction and semen abnormalities. Erectile dysfunction may be treated by the same therapies that are used in the general population. Similarly, the same treatments that are effective to assist conception in couples with non-SCI male factor patients are effective in assisting conception in SCI male-factor patients. The most apparent differences in male-factor symptoms between SCI and non-SCI patients are the high occurrences of anejaculation and atypical semen profiles in men with SCI. Methods available to assist ejaculation in men with SCI include penile vibratory stimulation and EEJ. Use of surgical sperm retrieval as the first line of treatment for anejaculation in men with SCI is controversial. Most men with SCI have a unique semen profile characterized by normal sperm concentration, but abnormally low sperm motility. Toxic substances in the semen contribute to this problem. Despite impaired sperm parameters, pregnancy outcomes using sperm from men with SCI are similar to pregnancy outcomes using sperm from non-SCI men. Future studies should focus on improving natural ejaculation and improving semen quality in these men.

## 1. Introduction

Spinal cord injury occurs most often to young men at the peak of their reproductive health [1]. In the United States, 80% of new injuries occur to men between the ages of 16 and 45 [2]. Similar statistics are found worldwide [3–12]. Owing to the fact that the most common causes of injury include motor vehicle accidents, violence, sport-related injuries, and falls, it has been assumed that the gender disparity is due to more men than women engaging risk-taking behavior that leads to injury. The actual reason for the disparity is unknown. There is some evidence suggesting that hormones, rather than behavior, may contribute to the disparity. For example, it has been shown that estrogen may be neuroprotective and/or that testosterone may be neurotoxic after injury [13, 14]. 

Following SCI, most men have severely impaired fertility characterized by erectile dysfunction (ED), ejaculatory dysfunction, and semen abnormalities [15–18]. This paper will discuss current treatments for infertility in men with SCI, including treatments for ED as well as methods of semen retrieval. A discussion of the latest research findings regarding causes of abnormal semen quality will also be presented. The paper will conclude with recommendations for treating infertile couples with a male partner with SCI. 

## 2. Treatment of Erectile Dysfunction (ED) in Men with SCI

The same treatments used for the management of erectile dysfunction in noninjured men are used for the management of ED in men with SCI. Because the basic mechanisms for erection (normal vasculature and an intact S2-S4 reflex arc) are preserved in most men with SCI, these men respond well to oral administration of phosphodiesterase-5 inhibitors (PDE-5 inhibitors), including Viagra (sildenafil citrate), Levitra (vardenafil HCL), and Cialis (tadalafil) [19–21]. Men with SCI who are poor responders to oral PDE-5 inhibitors may respond better to vasodilatory medications directly injected into the corpus cavernosum of the penis, such as alprostadil, Prostaglandin E-1 (PGE-1), or trimix, (a mixture of papaverine/Regitine/PGE-1). PGE-1 may also be given via an intraurethral suppository (MUSE). Nonpharmacologic management such as vacuum erection devices or implanted penile prostheses may also be offered. These treatments are discussed in more detail below.

### 2.1. PDE-5 Inhibitors

In the mid-1990's, sildenafil citrate, an agent being investigated as a coronary vasodilator, was reported to selectively inhibit PDE-5. PDE-5 inhibitors work by inhibiting the degradation of cyclic GMP (cGMP) by PDE-5. In the penile corpora, once an erection is initiated, cGMP is responsible for the maintenance of vascular smooth muscle relaxation, a critical activity necessary for the maintenance of erection. It should be noted that PDE-5 inhibitors do not initiate erections but act to maintain or improve erections. In 1998, the FDA approved sildenafil (Viagra) in the United States for the treatment of ED. In the same year, Derry et al. reported on the safety and efficacy of oral sildenafil in a group of patients with SCI [21]. These findings were confirmed in later studies in men with SCI [22, 23]. Other more recent PDE-5 inhibitors (vardenafil and tadalafil) were also found to be effective [24, 25]. 

All three of the current PDE-5 inhibitors report statistically significant improvement above baseline in all parameters measured such as responses to standardized questionnaires on erectile dysfunction. The percent of men with SCI who actually respond to the medicines varies from study to study and depends on what questions were asked and the dosages of the medicines used in each study. For example, sildenafil, the most studied of these agents, has the highest reported satisfaction rate, while tadalafil has the best reported results when measured 12–24 hours after ingestion. In general we can expect close to 70% of SCI men to respond to these oral agents. All of the agents share similar side effects, the most common being headache, flushing, heartburn, nasal stuffiness, and hypotension [26, 27].

### 2.2. Intracavernous Injections

Various agents, including papaverine, phentolamine, and alprostadil, have been administered intracavernosally for the purpose of treating erectile dysfunction [28, 29]. Currently, alprostadil is the only FDA-approved medicine for intracavernosal pharmacotherapy. The advantage of injecting alprostadil intracavernosally is that it works rapidly and is not dependent on the nitric oxide-PDE-5 system of maintaining high intracavernosal concentrations of cyclic GMP. Instead, alprostadil stimulates the production of cyclic AMP, another potent vasodilator within the corpora cavernosa. In men with SCI, there is an 80% success rate with this therapy; however, priapism is considered a major adverse effect [30–32]. Midodrine has been investigated as an adjunct therapy to mitigate priapism [33]. Other adverse side effects include hypotension, pain, and fibrosis. Patients with poor manual dexterity may require the assistance of a partner to perform the injections.

### 2.3. Intraurethral Application

Alprostadil, when formulated as a small suppository (MUSE), may also be administered intraurethrally. Reports of MUSE in patients with SCI have been disappointing. Undesirable outcomes included insufficient erections, pain in the urethra, and hypotension if administered without an accompanying constriction band placed at the base of the penis [34–36]. The absorption of alprostadil from the urethral lumen can be variable, especially in patients with SCI who manage their bladders with intermittent catheterization, which can reduce the effectiveness of MUSE. Some patients, however, may prefer MUSE because it is less invasive than intracavernosal injection. 

### 2.4. Vacuum Constriction Devices

Vacuum constriction devices (VCDs), also known as vacuum erection devices, have been used for decades to improve erections. Current devices work by inserting the penis into a rigid cylinder. A pump mechanism creates negative pressure to draw blood into the penis and create an erection. A constriction band is placed over the base of the penis to maintain erection.

In men with SCI, an erection considered satisfactory for intercourse was reportedly achieved in 90% of users [37, 38]. When used regularly, it is the most cost-effective method of treating erectile dysfunction. Although fairly safe and effective, the device detracts from the already compromised spontaneity of sexual relations, is unwieldy, and, for men with poor hand function, requires the assistance of a partner willing to participate in its use. Complications are usually related to the constriction band. For example, patients who are insensate in the genital area may be unable to detect pain associated with a forgotten constriction band, and this problem may result in anoxia of penile tissues.

### 2.5. Penile Prosthesis

The penile prosthesis was the first notable treatment for erectile dysfunction to become available for men with SCI. In the 1970s, experience accumulated on the use of penile prostheses for erectile dysfunction and/or for condom catheter stability in these men [39]. Long-term follow-up studies clearly showed that the prostheses produced satisfactory results in 60% to 80% of cases for erectile dysfunction, and over 90% of cases for urinary management. High complication rates (15% to 20%) were reported, especially in the earlier years with the use of noninflatable prostheses [40]. By contrast, inflatable prostheses in men with SCI showed lower complication rates [41–43]. For example, in one series of 28 patients, the perforation rate for the three-piece inflatable prosthesis was 0% [44]. 

### 2.6. Psychosocial Counseling

When treating ED, psychosocial counseling is recommended as an adjunct to pharmacological or device-oriented agents. When indicated, it is important to include the partner. An important goal of the counseling is to assist the patient or the couple in comprehending how a particular treatment works in the psychosocial context. Such therapy is important for the patient's compliance and motivation, the partner's acceptance, and the overall success of the treatment. 

## 3. Treatment of Ejaculatory Dysfunction

In the neurologically intact man, ejaculation is the result of coordination between psychological and physical sexual stimulation. The ejaculatory reflex is coordinated by the spinal cord and depends on thoracolumbar sympathetic fibers from segments T10-L2 and somatic fibers from segments S2-S4. This reflex receives its somatic input primarily from the dorsal nerve of the penis, which is activated by the stimulation of the glans penis. Neurons in the cortex, thalamus, hypothalamus, midbrain, and pons, all play a role in ejaculation; however, their exact functions are poorly understood [45, 46].

Spinal cord injury can disrupt the nerve pathways responsible for ejaculation. Only about 9% of men with SCI can ejaculate through masturbation or intercourse [47, 48]. Consequently, most men with SCI require some form of medical assistance to procure sperm for insemination.

### 3.1. Penile Vibratory Stimulation (PVS)

PVS is recommended as the first line of treatment for anejaculation in men with SCI because PVS is safe, reliable, and cost-effective and, compared to other methods, yields the highest number of total motile sperm in antegrade fractions [49–51]. PVS involves placing a vibrator on the dorsum or frenulum of the glans penis. Mechanical stimulation produced by the vibrator recruits the ejaculatory reflex to induce ejaculation [52]. PVS is more effective in men with an intact ejaculatory reflex, that is, men with a level of injury T10 or higher. Ejaculatory success requires an intact dorsal penile nerve terminating in sacral spinal cord segments S2-S4 [53].

A wide variety of devices can be used to perform PVS, but the most effective are devices delivering at least 2.5 mm of amplitude [54]. Two devices have been approved by the FDA for ejaculation of men with SCI, the Ferti Care personal and the Viberect. These devices became commercially available in 1995 and in 2011, respectively. In a study of 2,065 PVS procedures with the Ferti Care in 461 men with SCI, the ejaculation success rate was 86% in patients whose neurological level of injury was T10 or rostral. This rate dropped to 21% in patients whose level of injury was T11 or caudal [47]. Ejaculation success rates using the Viberect in men with SCI have not yet been reported.

The procedure of PVS has been well described in previous publications [52, 55]. Briefly, a vibrator is applied for 2-3 minutes or until antegrade ejaculation occurs. If no ejaculation occurs, stimulation is stopped for 1-2 minutes, and then resumed. These steps are repeated for up to 10 minutes of stimulation. Most patients (89%) who are responsive to PVS will ejaculate, however, within two minutes of stimulation onset [54].

If a patient is unable to ejaculate with the application of one Ferti Care vibrator, the penis may be sandwiched between two Ferti Care vibrators. This “sandwich technique” salvaged 22% of failures to one Ferti Care vibrator [56]. Other strategies have been reported to improve ejaculation success rates by PVS in men with SCI, including the use of abdominal electrical stimulation [57], oral administration of PDE-5 inhibitors [23], or oral administration of midodrine [58] prior to PVS. 

PVS can provoke autonomic dysreflexia, especially in patients whose level of injury is T6 or rostral [59]. Autonomic dysreflexia is caused by extensive sympathetic discharge due to a noxious stimulus originating below the level of injury [60, 61]. Autonomic dysreflexia can be managed by the administration of medications prior to the onset of PVS. A common medication for this purpose is nifedipine, a calcium channel blocker, administered sublingually in a dose of 20 mg, 15 minutes prior to PVS onset. Blood pressure must be monitored continuously throughout the PVS procedure. The angiotensin-converting enzyme inhibitor, captopril, 25 mg sublingual, has been reported to be effective in hypertension associated with autonomic dysreflexia and might be considered as an alternative to nifedipine [59, 62]. PDE-5 inhibitors should not be used in a situation in which the use of any potent blood-pressure-lowering medicine is anticipated.

### 3.2. Electroejaculation (EEJ)

Patients who cannot respond to PVS are often referred for EEJ, which must be administered by a physician trained in this procedure. The only FDA-approved device for performing EEJ is the Seager Electroejaculator (Dalzell Medical Systems, USA). The procedure of EEJ has been previously described [49, 63, 64], and is summarized here. Ejaculation is induced by placing the patient in the lateral decubitus position and inserting the probe into the rectum with the electrodes facing the seminal vesicles and prostate gland. Electric current is then delivered in a pattern of five seconds of stimulation followed by rest periods of approximately 20 seconds, during which ejaculation can occur. Ejaculation may occur in a dribbling, non projectile manner. It is important to milk the urethra to retrieve as much semen as possible. If no ejaculation occurs, current delivery is increased in 2 volt increments [63].

A study of sphincteric events during EEJ in men with SCI showed that when the current was turned off, the pressure differential between the internal and external urinary sphincters favored antegrade versus retrograde flow of semen [65]. This finding was later confirmed in a study showing a higher proportion of sperm in antegrade versus retrograde fractions of men with SCI when electricity during EEJ was delivered in an interrupted (as described above) versus continuous pattern [63]. 

Prior to EEJ, the bladder is prepared for retrograde ejaculation, which is common with EEJ [50, 51]. Bladder preparation includes draining urine by catheterization and instilling a buffering medium. After the procedure, the bladder is catheterized again to empty the retrograde fraction. As with PVS, EEJ can provoke autonomic dysreflexia, and blood pressure must be managed. The management regimen described for PVS may also be used for EEJ. 

The majority of men with SCI can undergo EEJ without anesthesia. For men with preserved pelvic sensation, EEJ can cause significant discomfort, and conscious sedation or general anesthesia may be necessary. If general anesthesia is required, the additional cost should be considered in the overall treatment plan for the couple [66]. EEJ is a highly successful procedure, with 95% resulting in ejaculation. The 5% failure rate can often be attributed to cases in which men with retained pelvic sensation experienced pain at low voltages (1–4 volts) on their first trial of EEJ and did not want to continue with further trials of EEJ under sedation or general anesthesia [67].

### 3.3. Prostate Massage (PM)

The name “prostate massage” is a misnomer because the seminal vesicles, as well as the prostate, are emptied during the procedure, which is performed by a physician who presses on these structures with a finger inserted into the patient's rectum. The rationale for performing PM is that sperm are stored in the ampulla of the vas deferens and, in men with SCI, are sequestered in the seminal vesicles as well [68]. The practitioner, therefore, attempts to mechanically push the sperm out through the ejaculatory ductal system.

PM has been successful in obtaining sperm from men with SCI and inducing pregnancy in their partner [69–71]. Sperm yields are typically low with PM compared to PVS or EEJ, therefore, it is not clear when PM is recommended in the algorithm of semen retrieval methods. In countries with easy access to EEJ; PM may be useful when PVS fails and the patient has retained pelvic sensation (i.e., the extra costs involved in performing EEJ under general anesthesia may be prohibitive) [66]. In countries without easy access to EEJ, PM may be useful when PVS fails.

### 3.4. Surgical Sperm Retrieval (SSR)

SSR is a method of retrieving sperm from reproductive tissue, either by aspiration or through surgical exploration. A variety of techniques may be used, including testicular sperm extraction (TESE), testicular sperm aspiration (TESA), microepididymal sperm aspiration (MESA), percutaneous epididymal sperm aspiration (PESA), and aspiration of sperm from the vas deferens [71–77]. Unlike PVS and EEJ, SSR was not developed to treat anejaculation. Instead, SSR was originally developed to retrieve sperm from men without SCI, who were azoospermic, that is, men who had no sperm in their ejaculate. In the algorithm of sperm retrieval methods in men with SCI, SSR should be performed only if PVS or EEJ fail or are not possible, as PVS and EEJ are less expensive, less invasive, and result in a higher motile sperm count. The low motile sperm count obtained with SSR commits the couple to intracytoplasmic sperm injection (ICSI), the most invasive and expensive of the currently available assisted reproductive technologies. For many couples, the cost of ICSI (US$8,000–12,000 per cycle) is prohibitive because it is usually not covered by medical insurance. 

Certain risks are associated with IVF-ICSI, such as complications resulting from superovulation, and the increased probability of multiple gestations with associated complications to the offspring. For the male partner, SSR procedures sometimes result in hematomas and pain. Collectively, these factors add increased costs and morbidity to the IVF-ICSI procedure [78]. Serious complications, including injury to the arteries and partial testicular infarction or permanent testicular devascularization, are rare [79, 80].

The application of SSR to men with SCI is controversial. A survey of IVF centers [67] indicated that some practitioners are using SSR as the first line of treatment for anejaculation in men with SCI. Practitioners cited a lack of equipment and/or a lack of education as reasons for not offering semen retrieval by PVS or EEJ. Ejaculation success rates, however, indicate that PVS and EEJ warrant consideration in centers not offering these options for couples with SCI male partners [47, 67]. The procedures of intravaginal insemination or intrauterine insemination (IUI) should likewise be considered because these methods have resulted in reasonable pregnancy rates in these couples [67, 81, 82]. 

## 4. Semen Quality in Men with SCI

The majority of men with SCI have a distinct semen profile characterized by normal total sperm count, but abnormally low sperm motility [55, 83, 84]. The cause of this condition is unknown and has been an area of active investigation. This section will review research on this topic. 

### 4.1. Role of Hormonal Alterations

Alterations in the hypothalamic-pituitary-gonadal axis may result in the disruption of spermatogenesis. The endocrine status of men with SCI has been examined in several studies which provided contradictory results. Some studies in humans [85–87] and animals [88, 89] have reported different hormone abnormalities associated with SCI, but no consistent correlation with the semen quality was shown. In a study of 66 men with SCI, no association was found between semen quality and serum levels of luteinizing hormone, follicle-stimulating hormone, testosterone, or prolactin [90]. The only exception was in a subgroup of subjects who had elevated levels of follicle-stimulating hormone. In each case, the patient was azoospermic, even patients with only small elevations of follicle-stimulating hormone. Hormonal alterations are unlikely to be a major contributor to poor semen quality in men with SCI.

### 4.2. Role of Scrotal Temperature

Spermatogenesis is a temperature-sensitive process and proceeds optimally at 35°C. Higher scrotal temperatures could have detrimental effects on sperm production [91]. It has been hypothesized that prolonged sitting in a wheelchair may elevate scrotal temperatures and lead to semen abnormalities in men with SCI [92]. Some studies showed that men with SCI sitting in wheelchairs had higher scrotal temperatures compared to able-bodied men, sitting in armchairs [86, 93]. One study found an inverse correlation between scrotal temperatures and motile sperm counts in men with SCI [93]. In contrast, another study found no difference between control subjects and SCI subjects in oral temperature, scrotal temperature, or the difference between these two parameters [90]. Furthermore, men with SCI who were ambulatory (i.e., not in wheelchairs) still had impaired semen quality [90], indicating that some aspect of the spinal cord injury, other than the simple act of sitting in a wheelchair, contributes to abnormal semen quality in these men. Supporting this idea is the fact that no study has found improvement in semen quality by cooling the scrotum of men with SCI. Studies of scrotal temperature in noninjured men have suggested that short-term versus long-term exposure to elevated temperature causes reversible versus irreversible changes in the seminiferous tubules [94, 95]. 

In men with SCI, however, both cross-sectional [96] and longitudinal [97] studies have shown that semen parameters were not significantly related to the duration of the postinjury period, suggesting a stable pattern for the measures across time. 

In light of these facts, it appears that no strong evidence exists to support the role of elevated scrotal temperature as a leading etiologic factor for the semen abnormalities in men with SCI.

### 4.3. Role of Bladder Management

Voiding dysfunction is commonly encountered in those with spinal cord injuries. The management of voiding dysfunction varies depending on the type of bladder and sphincter functions following SCI. Common management regimes include intermittent catheterization, indwelling urethral catheters, suprapubic catheterization, and spontaneous voiding. No bladder management regime has been associated with normal semen quality in men with spinal cord injury. However, some studies have shown that the use of intermittent catheterization is associated with better sperm motility than the use of the other methods [98, 99]. Although semen quality is improved with intermittent catheterization versus the other methods mentioned, it does not become normalized. Bladder management, then, does not seem to be a significant cause of impaired semen quality in men with SCI. 

### 4.4. Role of Ejaculation Frequency

The majority of men with SCI cannot ejaculate without medical assistance. It has been hypothesized that long periods between ejaculations may result in reproductive tract stasis which can negatively affect sperm. However, most studies investigating the effect of repeated ejaculation on semen quality in men with SCI found no improvement in semen parameters [72, 100–104]. These findings indicate that infrequency of ejaculation is not the sole factor causing abnormal semen quality in men with SCI.

### 4.5. Accessory Gland Function in Men with SCI

 In humans, semen is composed of fluids primarily from the seminal vesicles and prostate gland. The examination of semen from men with SCI shows numerous abnormalities in addition to abnormal sperm parameters. For example, 27% of men with SCI have brown-colored semen which does not become normally colored with repeated ejaculations [105]. The brown color is not simply hematospermia but, instead, indicates a dysfunction of the seminal vesicles [105]. Additional evidence of seminal vesicle dysfunction is the finding that men with SCI show an abnormal pattern of transport and storage of sperm in the seminal vesicles [68].

 In addition to dysfunction of the seminal vesicles in men with SCI, there is also an evidence of prostate gland dysfunction in these men. Prostate specific antigen (PSA) was higher in the blood, but lower in the semen of men with SCI compared to healthy, age-matched control subjects [106]. This pattern of PSA expression indicates a secretory dysfunction of the prostate gland in men with SCI. 

 Additional evidence of accessory gland dysfunction in men with SCI is found in studies showing abnormal concentrations of various biochemical substances in the semen of men with SCI compared to control subjects. For example, compared to able-bodied men, men with SCI have higher concentrations of platelet-acting factor acetylhydrolase (PAFah) [107], reactive oxygen species [108–110], and somatostatin (in patients with lesions at or above T6) [111]. Conversely, the semen of men with SCI has lower levels of fructose, albumin, glutamic oxaloacetic transaminase, alkaline phosphatase, and TGF-beta 1 compared to the semen of able bodied men [112, 113].

### 4.6. Seminal Plasma from Men with SCI Is Toxic

Evidence of abnormal accessory gland function in men with SCI led to studies investigating the role of the seminal plasma as a contributing factor to the abnormal sperm parameters found in these men. The studies showed that the seminal plasma of men with SCI is toxic to normal sperm. For example, when seminal plasma of men with SCI was mixed with sperm from normospermic men, a rapid and profound impairment to normal sperm motility occurred [114]. Furthermore, sperm unexposed to the seminal plasma (i.e., aspirated from the vas deferens) had significantly higher motility than sperm in the ejaculate of these men [115]. These findings introduced the concept of an abnormal seminal plasma environment as a cause of impaired sperm motility in men with SCI. 

### 4.7. Men with SCI Have Leukocytospermia

One of the most pronounced abnormalities in men with SCI is leukocytospermia, which is an abnormally high concentration of white blood cells in the semen [116–118]. Leukocytospermia has been studied in non-SCI men, especially with respect to its relationship with genitourinary tract infections and infertility [119]. These studies have established that cellular elements, in general, may be related to abnormal sperm parameters [120–123], but the sperm-leukocyte interaction is not clearly understood [122, 124]. Low sperm motility in men with SCI does not seem to be caused simply by local infection of the genitourinary tract. In these men, treatment of genitourinary infections with antibiotics does not result in improved sperm motility [119].

### 4.8. Men with SCI Have Immune Abnormalities

There is ample experimental evidence that individuals with SCI suffer from immune regulatory dysfunction [87, 125–127]. Typically, their circulating lymphocytes demonstrate suppressed responses to challenges that stimulate cell division (standard mitogen challenges), have reduced ratios of specific white blood cells, and show reduced natural killer (NK) cell responses and altered response to exercise challenges. The conclusion of these studies is that autonomic nervous system dysfunction results in alterations of the normal operations of the immune response possibly via the interruption of sympathetic innervation of the lymphatics and spleen, the normal hypothalamic-pituitary-adrenal axis, or normal neurologic feedback from the periphery on these systems. The relationship of these findings to any disease state is unclear. In examining the semen of men with SCI during routine semen analysis, nearly all have an elevated number of white blood cells (WBCs) [116]. Flow cytometric analysis of the semen of these men has shown the presence of large numbers of activated T lymphocytes [116]. Activated T lymphocytes are known to secrete cytotoxic cytokines [128]. It is well known that activated T lymphocytes can exert a damaging effect on other cells by cytotoxic cytokines [128–131].

Cytokines play an important role in the function of the immune system [128]. Elevated concentrations of cytokines can be harmful to sperm [132–134]. It is possible that the activated T lymphocytes observed in semen of men with SCI are secreting cytokines which impair sperm motility. It is hypothesized that semen cytokine concentrations are abnormal in men with SCI. Basu et al. [113] measured levels of ten cytokines in the seminal plasma of men with SCI versus age-matched, healthy, and non-SCI control subjects. The results showed that, compared to control subjects, concentrations of five of the ten cytokines were elevated in the seminal plasma of men with SCI [113]. Further, interfering with the actions of specific cytokines, by the addition of monoclonal antibodies directly to the semen, improved sperm motility in men with SCI [113, 135]. This treatment represented the first intervention that significantly improved sperm motility in men with SCI. 

The mechanism leading to elevated semen cytokines in men with SCI is unknown. We investigated the inflammasome signaling mechanism as a contributor to this problem. The inflammasome is a multiprotein complex responsible for activating the innate immune response [136, 137]. Semen of men with SCI versus semen of healthy control subjects was investigated for two important components of the inflammasome: ASC and caspase 1. Our study found that these components were increased in semen of men with SCI relative to semen of control subjects and that these proteins were correlated with increased expression of the pro inflammatory cytokines IL-1*β* and IL-18. These findings suggested a role of the inflammasome complex in abnormal semen quality in men with SCI [138]. 

## 5. Reproductive Options for Couples with SCI Male Partners

### 5.1. Intravaginal Insemination at Home

 The majority of men with SCI cannot ejaculate during sexual intercourse and require some form of technical or medical assistance to father a child. The least invasive and least expensive of the assisted reproductive options is intravaginal insemination, sometimes called “in-home insemination.” It is advisable that couples be evaluated in a clinic prior to attempting intravaginal insemination at home. The clinic should evaluate the male partner to determine the optimal method for safe and effective ejaculation at home. This evaluation should assess the male partner with SCI for risk of, and management of, autonomic dysreflexia. The evaluation should also determine the optimal method of inducing ejaculation, such as the use of one vibrator [52] two vibrators [56], abdominal electrical stimulation plus PVS [57], or oral medications, such as Viagra, prior to PVS [25]. The clinic should also evaluate the semen quality of the male partner with SCI. While minimum numbers of total motile sperm have not been established for successful pregnancy using intravaginally inseminated sperm from men with SCI, the clinic should discuss guidelines regarding the number of intravaginal insemination cycles that will be attempted prior to choosing more advanced methods of assisted conception.

 The female partner should be evaluated for the absence of any tubal or uterine pathology and for the presence of normal ovulatory cycles. She should also be counseled regarding methods of ovulation prediction at home. Insemination should occur at the time of ovulation. If the male partner with SCI cannot ejaculate during intercourse, the couple may collect his semen by PVS into a clean specimen cup. The semen is then drawn into the barrel of a syringe, which may then be used to deposit the semen into the vagina. 

 Good pregnancy rates have been achieved in couples with SCI male partners who attempted home intravaginal insemination. In a report of 45 such couples, 20 pregnancies were achieved in 17 couples (37.8% pregnancy rate) [81]. In a multicenter study from three countries, 82 pregnancies were achieved in 60 of 140 couples (43% pregnancy rate) [82]. 

### 5.2. Intrauterine Insemination (IUI)

 IUI has been used to achieve pregnancy in couples with a male partner with SCI. IUI involves collecting semen from the SCI male partner and processing it in a laboratory to separate the sperm from the semen, and to isolate the motile from the nonmotile sperm. In men with SCI, semen to be used in IUI is usually collected by PVS or EEJ. The processed sperm is placed inside the uterus of the woman. IUI can be performed during unstimulated cycles where no fertility drugs are prescribed to the woman or during stimulated cycles where fertility drugs are prescribed to stimulate the production of eggs and/or to stimulate ovulation. 

 Ohl et al. [66] studied 121 consecutive couples who used EEJ in combination with assisted reproductive technology in the treatment of anejaculatory infertility. For those couples that did not conceive within 3–6 cycles of IUI, gamete intrafallopian transfer (GIFT) or in vitro fertilization (IVF) procedures were recommended. Eighty-seven of the 121 couples had male partners with SCI. In 479 cycles of EEJ with IUI in these couples, 41 pregnancies were obtained. This outcome represents an 8.6% pregnancy rate per cycle and 32.2% pregnancy rate per couple.

 Ohl et al. [66] concluded that the type of fertility drug used to stimulate egg production and/or ovulation, and the method of monitoring and timing of insemination, did not affect IUI cycle fecundity. No multiple gestations were observed with natural cycle/IUI procedures. In comparing ranges of motile sperm counts and IUI cycle fecundity, the authors suggested that clients with counts of <4 million total motile sperm should proceed directly to high-level assisted reproductive technologies, since below this threshold the pregnancy rate per cycle decreased sharply to 1.1%. Based on cost-effectiveness estimation between IUI and IVF, they recommended that couples should attempt 3–6 cycles of IUI before proceeding to IVF. When inseminated total motile sperm counts were greater than 40 million, the pregnancy rate per cycle was 17.6%. They concluded that an IUI program can be successful and costeffective in men with SCI. 

 Kathiresan et al. reported on 57 couples with SCI male partners who underwent IUI. Fourteen couples achieved pregnancy (24.6% pregnancy rate), and there were a total of 19 pregnancies [81]. Previous studies have reported pregnancy rates ranging between 26.7% and 78.6% [139–146]. Taken together, these studies indicate that IUI is a reasonable option in this patient population, and that IUI warrants consideration before proceeding to in vitro fertilization.

### 5.3. In Vitro Insemination (IVF)/Intracytoplasmic Sperm Injection (ICSI)

 Advanced assisted reproductive technologies are available when fertilization is not possible or not indicated by intravaginal insemination or IUI. IVF is a procedure where sperm from the man are placed in a laboratory dish with ova retrieved from the woman. The sperm-ova mixture is then placed in an incubator for up to 5 days. Sperm are allowed to fertilize the ova. Embryos that develop to the highest quality blastocyst stage are then placed into the uterus of the woman. Transfer of high quality blastocysts are associated with higher pregnancy rates compared to transfer of poorly formed blastocysts [147]. When the number of motile sperm is too low for conventional IVF, the method of intracytoplasmic sperm injection (ICSI) is often used to achieve fertilization. ICSI is a procedure in which a single sperm is injected directly into the egg.

 IVF and ICSI have been used to achieve pregnancy in couples with male partners with SCI [140, 141, 144, 146, 148–151]. Similar pregnancy and live birth rates have been reported in couples with SCI male partners undergoing ICSI versus couples with other etiologies of male factor infertility. For example, Kathiresan et al. reported on 31 couples with SCI male partners who underwent 48 cycles of IVF/ICSI versus 297 couples with non-SCI male factor infertility who underwent 443 cycles of IVF/ICSI. The pregnancy rate per couple in the SCI group was 58.1% versus 57.9% in the non-SCI group 53.5%. This difference was not statistically significant. The live birth rate per couple in the SCI group was 51.6% versus 53.5% in the non-SCI group. This difference was not statistically significant. In the SCI group, sperm collected by PVS yielded similar IVF/ICSI success rates as sperm collected by EEJ [152]. 

 Other studies have confirmed these findings for couples with SCI versus non-SCI male partners undergoing IUI, IVF, and ICSI [140, 150, 153]. Although there are some studies showing impaired sperm function in men with SCI [83, 154], these functional impairments apparently do not lower pregnancy rates in couples. These findings may reflect the increasing ability of laboratory-assisted reproductive technologies to overcome all forms of male infertility [155, 156].

## 6. Conclusions

 Young men comprise the overwhelming majority of individuals with SCI. Fertility is severely impaired in men with SCI due to ED, ejaculatory dysfunction, and semen abnormalities. The same treatments that are effective for ED in the general population are effective for treatment of ED in the SCI population. Similarly, the same treatments that are effective to assist conception in couples with non-SCI male-factor patients are effective in assisting conception in SCI male-factor patients.

 The most apparent differences in male-factor symptoms between SCI and non-SCI patients are the high occurrences of anejaculation and atypical semen profiles in men with SCI. Methods available to assist ejaculation in men with SCI include PVS and EEJ. Use of SSR as the first line of treatment for anejaculation in men with SCI is controversial. 

 Most men with SCI have a unique semen profile characterized by normal sperm concentration, but abnormally low sperm motility and viability. Abnormal accessory gland function, possibly due to dysinnervation and/or other factors, may lead to altered seminal plasma that is toxic to sperm. Despite impaired sperm parameters, pregnancy outcomes using sperm from men with SCI are similar to pregnancy outcomes using sperm from non-SCI men. Future studies should focus on improving natural ejaculation, and improving semen quality in men with SCI. 
